# Comparison of Symptom Severity and Progression in Advanced Cancer Patients Among Different Care Settings: A Secondary Analysis

**DOI:** 10.1089/pmr.2023.0011

**Published:** 2023-06-23

**Authors:** Ryuto Shiraishi, Yoshiyuki Kizawa, Masanori Mori, Isseki Maeda, Yutaka Hatano, Hiroto Ishiki, Tomofumi Miura, Naosuke Yokomichi, Maiko Kodama, Keiko Inoue, Sen Otomo, Takashi Yamaguchi, Jun Hamano

**Affiliations:** ^1^ Department of Palliative Medicine, Kobe University School of Medicine, Kobe, Japan.; ^2^ Division of Clinical Medicine, Department of Palliative and Supportive Care, Faculty of Medicine, University of Tsukuba, Ibaraki, Japan.; ^3^ Palliative Care Team, Seirei Mikatahara General Hospital, Hamamatsu, Japan.; ^4^ Department of Palliative Care, Senri-Chuo Hospital, Toyonaka, Japan.; ^5^ Department of Palliative Care, Daini Kyoritsu Hospital, Kawanishi, Japan.; ^6^ Department of Palliative Medicine, National Cancer Center Hospital, Tokyo, Japan.; ^7^ Department of Palliative Medicine, National Cancer Center Hospital East, Kashiwa, Japan.; ^8^ Division of Palliative and Supportive Care, Seirei Mikatahara General Hospital, Hamamatsu, Japan.; ^9^ Orange Home-Care Clinic, Fukui, Japan.; ^10^ Medical Corporation Aisei-kai, Hirakata, Japan.; ^11^ Seimeikan Clinic, Sapporo, Japan.; ^12^ Division of Clinical Medicine, Faculty of Medicine, University of Tsukuba, Ibaraki, Japan.

**Keywords:** advanced cancer patients, multicenter prospective cohort studies, palliative care at home, palliative care units, place of care, symptom management

## Abstract

**Background:**

Most people in Japan wish to spend their final days at home, but the majority fail to do so; earlier studies indicated a more pronounced worsening of symptoms if treated at home.

**Objectives:**

This study compared the prevalence of symptom worsening and explored associated factors between patients with advanced cancer receiving palliative care in palliative care units (PCUs) and at home.

**Design:**

We conducted a secondary analysis of two multicenter, prospective cohort studies involving patients with advanced cancer receiving palliative care in PCUs or at home.

**Setting/Subjects:**

One study was conducted at 23 PCUs (January to December 2017) and the other on 45 palliative home care services (July to December 2017) in Japan.

**Measurements:**

Symptom changes were categorized as stable, improved, or worse.

**Results:**

Of the 2998 registered patients, 2877 were analyzed. Among them, 1890 patients received palliative care in PCUs, and 987 at home. Patients receiving palliative care at home were more likely to have worsening of pain (17.1% vs. 3.8%; *p* < 0.001) and drowsiness (32.6% vs. 22.2%; *p* < 0.001) than those in PCUs. By multivariate logistic regression analysis, palliative care at home was significantly associated with worsening of the Palliative Prognostic Index dyspnea subscale in the unadjusted model (odds ratio, 1.42 [95% confidence interval, 1.08–1.88]; *p* = 0.014) but not for any symptoms in the adjusted model.

**Conclusions:**

After adjusting for patient background, the prevalence of symptom worsening was not different between patients with advanced cancer receiving palliative care at home and in PCUs.

## Introduction

In any care setting, symptom management in the last days before death is important for patients and their families.^1–3^ Terminally ill patients need careful symptom management, and their families may need support and coaching as death approaches.^4^ Palliative care improves symptom control, satisfaction, and psychological support for patients and their families in palliative care units (PCUs) and at home, particularly at the end of life.^5^

The prevalence of symptoms in patients with advanced cancer varies substantially among previous studies,^6–10^ and the results about the degree of symptom relief in the last week of life are inconsistent. These discrepancies may be explained by differences in the care setting (PCU, hospice, outpatient pain clinic, or home), patient background, study design (prospective, retrospective, or cross-sectional study), measurement tools, and cancer type.^6–10^

Receiving care in a preferred place has a significant impact on a patient's quality of life.^11^ Currently, more than half of the patients worldwide prefer to be cared for and die at home, and spending the final days at home has a higher quality of life than in the hospital.^2,12–15^ In Japan, roughly 70% of citizens would like to die at home,^16^ but in fact, only ∼16% die at home.^17^

Barriers to staying at home at the end of life for patients with advanced cancer include anxiety about disease progression, inadequate explanation by the physician regarding treatment and medical condition, and insufficient symptom management.^18^ Therefore, we questioned whether symptom severity was different between patients receiving palliative care at home and in PCUs, where the quality of symptom management is generally considered to be better.

We found only one observational study that compared the change in symptom intensity between different care settings, that by Eagar et al.^5^ In that study, changes in symptoms in patients receiving palliative care in PCUs were explored and compared with those in patients receiving palliative care at home. When comparing all symptom outcomes by place of death, they found that patients receiving palliative care in PCUs were 3.7 times more likely to have no severe symptoms, compared with those at home. Patients receiving palliative care at home had less improvement overall and experienced worse fatigue and dyspnea.

However, we believe that their study had several limitations: (1) it did not adjust for patient background associated with symptom intensity, such as disease stage and estimated prognosis; and (2) patients in good general condition and with longer estimated survival tended to be treated at home more often in the first place,^19^ suggesting a bias.

In addition, the outcomes of symptom distress in that study were defined as the change in the number of severe symptoms from the start to the end of an episode of care, which may not be individualized in terms of the degree of symptom relief.^5^ Given these limitations, it is difficult to conclude from the results of that study alone that symptom management in home care is inferior to that in PCUs.

To overcome these limitations, the present study primarily aimed at comparing the prevalence of symptom worsening from admission to three days before death between patients with advanced cancer receiving palliative care in PCUs and those at home in Japan. Our secondary aim was to explore the factors associated with the worsening of symptoms during the period that patients received specialist palliative care.

## Materials and Methods

We conducted a secondary analysis of two multicenter, prospective cohort studies involving patients with advanced cancer receiving palliative care in PCUs (East Asian collaborative cross-cultural Study to Elucidate the Dying process [EASED] study) or at home (Come Home study) in Japan to compare the prevalence of symptom worsening. Both studies aimed at identifying the symptoms and medical treatment of these patients at the end of life.

The EASED study was conducted at 23 PCUs between January and December 2017,^20^ and the Come Home study was conducted on 45 palliative home care services between July and December 2017 in Japan.^21^

Palliative care specialists in PCUs and primary care physicians with expertise in palliative care at home were primarily responsible for each patient assessed, and they recorded all measurements on the day of registration. The physician assessed the patients at least once a day in PCUs, at least once a week at home, and often every day. In both studies, the physicians usually assessed and recorded symptoms and treatments at every visit, but in some cases, they assessed and recorded retrospectively from medical records and memory after the observation period.

They followed the patients until death or for six months after registration. The observation period ended when patients were discharged from PCUs or palliative home care, or at death.

### Participants

In the EASED study, patients were enrolled when they were admitted to PCUs, whereas in the Come Home study, patients were enrolled when they started receiving palliative care at home from the participating facilities during the study period. Both studies had the following eligibility criteria: (1) 18 years old or older; (2) locally advanced or metastatic cancer (including hematopoietic neoplasms); and (3) started receiving palliative care in PCUs or at home at the participating facilities.

All patients who refused to participate in either of these studies were excluded. In addition, patients scheduled to be discharged or transferred within a week of admission to PCUs were excluded from the EASED study.

### Measurements

The severity of symptoms, such as pain, shortness of breath, weakness or lack of energy, sore or dry mouth, and drowsiness, were assessed by the responsible physician using the Integrated Palliative Care Outcome Scale (IPOS) Staff version in Japanese^22^ and scored as 0 (not at all), 1 (slightly), 2 (moderately), 3 (severely), and 4 (overwhelmingly).

The IPOS is a rational outcome measure developed to comprehensively evaluate physical symptoms, psychological state, and spiritual needs^23,24^ and it is currently used as a standardized measure worldwide. The Japanese version has already been confirmed to be valid and reliable.^22,25^ The dyspnea subscale of the Palliative Prognostic Index (PPI)^26^ was also used to assess shortness of breath, and scored as 0 (no dyspnea), 1 (dyspnea only on exertion), and 2 (dyspnea even at rest) during assessment.

Given that the IPOS (shortness of breath) was only assessed on admission, we considered it insufficient to compare the degree of symptom relief. Hence, we assessed symptom severity on admission and at three days before death. To adjust for background factors that might affect symptom severity during assessment, we collected some other data on the day of registration.

These data, which were obtained from previous studies and discussions among researchers,^5,27–29^ included age, sex, metastatic site (liver, bone, lung, and central nervous system), age-adjusted Charlson Comorbidity Index (ACCI),^30^ opioid dosage (oral morphine equivalent [OME]),^28^ and data used for formulating the Prognosis in Palliative Care Study predictor models-A (PiPS-A).^31^

The modified PiPS-A consists of the following: primary cancer site; metastasis site; Abbreviated Mental Test score judged by the physicians; pulse rate; anorexia; dyspnea, dysphasia; fatigue; weight loss in the previous month; Eastern Cooperative Oncology Group performance status (ECOG PS); and global health status (rated on a specific 7-point scale used in the original study, with 1 as “extremely poor health” and 7 as “normal health”).

Symptoms were recorded as being either present or absent. Cognitive status was assessed according to the Abbreviated Mental Test score used in the original model of the Prognosis in Palliative Care Study reported by Gwilliam et al.^32^ In the present study, cognitive status was rated as absent if the Abbreviated Mental Test score was above three points or as present if the score was 3 or below (the physician conducted the scoring without interviewing the patient).

We also collected data on the day of registration to formulate the PPI. The PPI consists of the following: Palliative Performance Scale (classified into three groups; 10–20, 30–50, and ≥60); oral intake (classified as severely reduced, moderately reduced, or absent); edema (classified as present or absent); dyspnea at rest (present or absent); and delirium (present or absent), defined by the Diagnostic and Statistical Manual of Mental Disorders 5 (DSM-5).

The demographic and clinical characteristics of participants were also collected on the day of registration. These characteristics included the primary cancer site, bowel obstruction (classified as present or absent), pleural effusion (present or absent), ascites (present or absent), chemotherapy within a month, oxygen therapy use, antipsychotic use, and psychotropic use. Likewise, we collected several other data on treatment before death, such as opioid dosage and parenteral hydration at one week before death.

### Outcomes

The prevalence of symptoms was defined as the rate of patients who scored two to four points in the IPOS assessment based on previous studies.^22,33^ We also defined the prevalence of dyspnea as the rate of patients who scored two points on the PPI dyspnea subscale. In addition, the changes in symptoms between admission and at three days before death were defined as stable, improved, or worse (no change, improvement by ≥1 point, and deterioration by ≥1 point, respectively).^34^

### Statistical analysis

First, patients with an unknown date of death were excluded from the analysis, although we did not exclude or separate patients who moved from one setting to another. Then, we conducted descriptive analyses of the demographic characteristics. Using the chi-squared test or Fisher's exact test, we compared the prevalence of pain, shortness of breath, weakness or lack of energy, sore or dry mouth, and drowsiness on admission and at three days before death between patients receiving palliative care in PCUs and those at home.

Changes in symptoms between admission and at three days before death were compared using the chi-squared test (Primary aim).^34^ We treated the IPOS of cannot assess as “cannot assess” in the descriptive analyses and as “missing values” when evaluating the changes in symptoms. We then conducted a univariate logistic regression analysis of the changes in symptoms from the time of admission to three days before death between the two groups.

We next applied multivariate multiple imputations by chained equations to patients with missing data before death and those who moved from home to other settings, and then compared the worsening of symptoms during the period patients received specialist palliative care.^35^

The imputation process created 10 complete datasets with the following covariates: severity of all symptoms on admission and at three days before death; age; sex; primary lung cancer; metastasis site (bone, lung, and central nervous system); anorexia; weight loss in the previous month; pleural effusion; delirium; modified PiPS-A; PPI; ACCI; opioid dosage; chemotherapy within a month; oxygen therapy use; antipsychotic use; and psychotropic use at registration; opioid dosage; and parenteral hydration one week before death.

To adjust for patient background, symptoms, and treatment that might affect symptom severity from admission to three days before death, we conducted a multivariate logistic regression analysis. All factors affecting the worsening of symptoms were determined according to previous studies and discussions among researchers.^5,27–29,36^

Independent variables, such as the place of care, age (≥65 years), sex, modified PiPS-A, PPI, ACCI, opioid dosage at registration, and opioid dosage at one week before death, were possible factors affecting changes in all five symptoms. Specific independent variables were, for example, bone metastasis at registration for factors affecting changes in pain, pleural effusion at registration for shortness of breath, anorexia at registration for weakness or lack of energy, oxygen therapy use at registration for sore or dry mouth, and delirium at registration for drowsiness.

The PPI (≥6.5), ACCI (≥6), and opioid dosage (OME ≥60 mg/day) were categorized as binary variables based on previous studies,^26,28,37,38^ and only the parenteral hydration was categorized into three variables (0, 1–999, and ≥1000 mL/day) according to previous studies.^36,39^ A *p*-value <0.05 was considered to indicate a significant difference. All statistical data were analyzed using the SPSS-J software (version 27.0; IBM, Tokyo, Japan).

### Ethics

Both studies conformed to the ethical standards of the Declaration of Helsinki and the ethical guidelines for research provided by the Ministry of Health, Labor and Welfare in Japan. The present study was approved by the institutional review boards of all participating facilities. Further, the use of existing data for secondary analysis and their combination were approved by the main institutional review boards (PCUs: Seirei Mikatahara General Hospital [Research No. 16–29]; palliative home care: University of Tsukuba [Research No. 1153]).

## Results

**FIG. 1.**
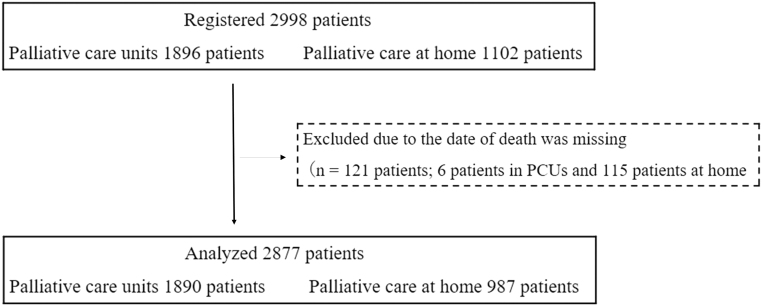
Participants' flow diagram. PCUs, palliative care units.

Out of the 2998 patients registered in both studies, 1896 were admitted to PCUs and 1102 received palliative care at home. However, 121 patients were excluded because the date of death was missing (6 in PCUs and 115 at home). Ultimately, 2877 patients were analyzed, with 1890 in PCUs and 987 at home (Fig. 1).

The mean age was 72.5 years (95% confidence interval, 72.1–73.0), and 52.7% were men. The most common sites of primary cancer were the gastrointestinal tract/hepatobiliary system and pancreas (48.0%), followed by the lungs (17.3%). Around 80% of patients were estimated to have a prognosis of surviving days or weeks using the modified PiPS-A (Table 1).

**Table 1.** Patient Characteristics at Registration

*Variables*	*All patients (***n*** = 2877)*		*PCUs (***n*** = 1890)*		*Palliative care at home (***n*** = 987)*		****p****
	****n****	*%*	****n****	*%*	****n****	*%*	
Age ≥65	2250	78.2	1457	77.1	793	80.3	0.045
Male sex	1517	52.7	960	50.8	557	56.4	0.004
Married	1850	64.3	1150	60.8	700	70.9	<0.001
Living with family	2224	77.3	1373	72.6	851	86.2	<0.001
With underage child	118	4.1	74	3.9	44	4.5	0.524
Site of primary cancer							
Lung	499	17.3	318	16.8	181	18.3	0.059
Gastrointestinal/hepatobiliary and pancreas	1380	48.0	886	46.9	494	50.1	
Breast	184	6.4	131	6.9	53	5.4	
Gynecological	180	6.3	119	6.3	61	6.2	
Urogenital	220	7.6	141	7.5	79	8.0	
Others	414	14.4	295	15.6	119	12.0	
Site of metastasis							
Anywhere	2326	80.8	1603	84.8	723	73.3	<0.001
Liver	1066	37.1	729	38.6	337	34.1	0.023
Bone	713	24.8	500	26.5	213	21.6	0.005
Lung	980	34.1	707	37.4	273	27.7	<0.001
Central nervous system	371	12.9	263	13.9	108	10.9	0.025
Anorexia	2367	82.3	1550	82.0	817	82.8	0.651
Dysphagia	823	28.6	622	32.9	201	20.4	<0.001
Weight loss in the previous month	2159	75.0	1380	73.0	779	78.9	<0.001
Edema	1243	43.2	869	46.0	374	37.9	<0.001
Bowel obstruction	355	12.3	256	13.5	99	10.0	0.007
Pleural effusion	748	26.0	554	29.3	194	19.7	<0.001
Ascites	851	29.6	567	30.0	284	28.8	0.494
Delirium (DSM-5)	677	23.5	582	30.8	95	9.6	<0.001
Abbreviated Mental Test by physician judging ≤3	841	29.2	672	35.6	169	17.1	<0.001
ECOG PS							
0–1	128	4.4	24	1.3	104	10.5	<0.001
2	377	13.1	157	8.3	220	22.3	
3	1167	40.6	795	42.1	372	37.7	
4	1205	41.9	914	48.4	291	29.5	
Modified PiPS-A							
Months	508	17.7	251	13.3	257	26.0	<0.001
Weeks	1428	49.6	893	47.2	535	54.2	
Days	908	31.6	722	38.2	186	18.8	
Palliative Prognostic Index ≥6.5	905	31.5	738	39.0	167	16.9	<0.001
Age-adjusted Charlson comorbidity index ≥6	2752	95.7	1827	96.7	925	93.7	0.001
Opioid dosage (OME ≥60 mg/day)	614	21.3	450	23.8	164	16.6	<0.001
Chemotherapy within a month	370	12.9	172	9.1	198	20.1	<0.001
Oxygen therapy use	704	24.5	568	30.1	136	13.8	<0.001
Antipsychotic use	526	18.3	427	22.6	99	10.0	<0.001
Psychotropic use	824	28.6	557	29.5	267	27.1	0.170
	**Mean**	**95% CI**	**Mean**	**95% CI**	**Mean**	**95% CI**	
Age (years)	72.5	72.1–73.0	72.4	71.8–72.9	72.8	72.1–73.5	0.351
Age-adjusted Charlson comorbidity index	10.8	10.7–10.9	11.0	10.8–11.1	10.5	10.3–10.6	<0.001
Palliative Prognostic Index	5.4	5.3–5.5	6.1	5.9–6.2	4.1	3.9–4.3	<0.001
Opioid dosage per day (OME, mg/day)	42.0	36.1–47.8	43.4	39.6–47.3	39.1	23.6–54.6	0.495
Survival (days)	35.2	33.6–36.7	26.9	25.6–28.3	51.0	47.5–54.4	<0.001

CI, confidence interval; DSM, Diagnostic and Statistical Manual of Mental Disorders; ECOG PS, Eastern Cooperative Oncology Group performance status; OME, oral morphine equivalent; PCUs, palliative care units; PiPS-A, Prognosis in Palliative Care Study predictor model A.

### Comparison of the prevalence of severe symptoms on admission and at three days before death

On admission, symptoms that had a significantly higher prevalence in the PCU group were dyspnea (PPI), sore or dry mouth, and drowsiness, whereas no symptoms had a higher prevalence in the home group. At three days before death, symptoms that had a significantly higher prevalence in the PCU group were dyspnea (PPI) and weakness or lack of energy, whereas pain was a symptom that had a significantly higher prevalence in the home group (Table 2).

**Table 2.** Prevalence of Severe Symptoms on Admission and at Three Days Before Death Between Palliative Care Units and Palliative Care at Home

*Variables*		*All patients*		*PCUs*		*Palliative care at home*		****p****
		****n****	*%*	****n****	*%*	****n****	*%*	
Pain IPOS ≥2	On admission (*n* = 2765)	1023	37.0	664	37.1	359	36.8	0.886
	Three days before death (*n* = 1097)	319	29.1	90	19.0	229	36.8	<0.001
Shortness of breath IPOS ≥2	On admission (*n* = 2766)	590	21.3	380	21.2	210	21.5	0.860
Dyspnea subscale of the PPI = 2	On admission (*n* = 2876)	438	15.2	344	18.2	94	9.5	<0.001
	Three days before death (*n* = 2310)	610	26.4	464	28.4	146	21.5	0.001
Weakness or lack of energy IPOS ≥2	On admission (*n* = 2752)	1198	43.5	785	44.2	413	42.4	0.376
	Three days before death (*n* = 1680)	984	58.6	648	60.9	336	54.5	0.011
Sore or dry mouth IPOS ≥2	On admission (*n* = 2751)	503	18.3	364	20.5	139	14.3	<0.001
	Three days before death (*n* = 1680)	546	32.5	360	33.9	186	30.1	0.117
Drowsiness IPOS ≥2	On admission (*n* = 2752)	594	21.6	413	23.2	181	18.6	0.004
	Three days before death (*n* = 1676)	703	41.9	436	41.1	267	43.4	0.353

IPOS, integrated palliative care outcome scale; PPI, palliative prognostic index.

Opioid dosage (OME ≥60 mg/day) was significantly more prevalent at one week before death in patients in PCUs than in those who received palliative care at home (24.1% vs. 18.2%; *p* < 0.001). The patients in PCUs received significantly more parenteral hydration at one week before death than patients receiving palliative care at home (0 mL/day, 38.4% vs. 65.2%; 1–999 mL/day, 40.8% vs. 11.4%; ≥1000 mL/day, 7.1% vs. 2.6%; *p* < 0.001).

### Changes in symptoms from admission to three days before death

**FIG. 2.**
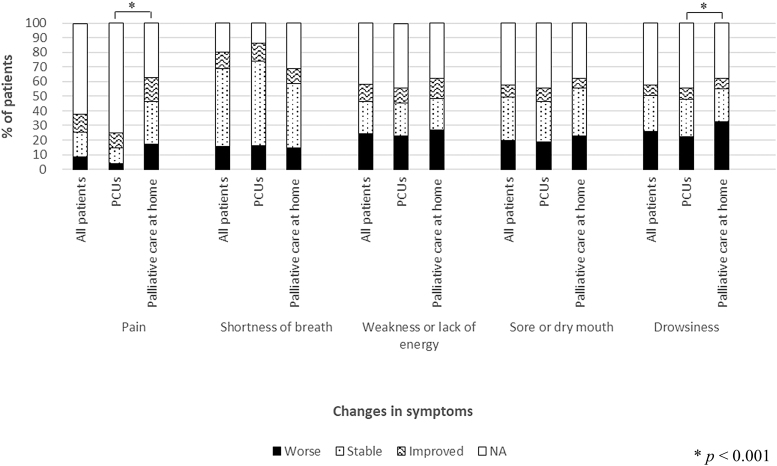
Changes in symptoms from admission to three days before death. The changes in symptoms were defined as stable, improved, or worse (no change, improvement by ≥1 point, and deterioration by ≥1 point, respectively). The *p*-values were derived from the chi-squared test (Primary aim).

Patients who received palliative care at home were significantly more likely to have worsening of pain (17.1% vs. 3.8%; *p* < 0.001) and drowsiness (32.6% vs. 22.2%; *p* < 0.001) than those in PCUs. No significant differences were found in the proportion of patients who experienced worsening of dyspnea (PPI, 14.8% vs. 16.0%; *p* = 0.095), weakness or lack of energy (26.8% vs. 22.8%; *p* = 0.365), and sore or dry mouth (22.6% vs. 18.6%; *p* = 0.235) between palliative care at home and PCUs (Fig. 2).

### Multivariate logistic regression analysis of factors associated with increased symptom prevalence

The prevalence of patients with worsening of the following five symptoms (pain, shortness of breath, weakness or lack of energy, sore or dry mouth, and drowsiness) was not different between palliative care at home and in the PCU in the adjusted model using multivariate logistic regression analysis. The factors that showed a significant positive association with dyspnea (PPI) worsening were primary lung cancer (odds ratio [OR], 1.48; *p* = 0.020), lung metastases (OR, 1.41; *p* < 0.001), and pleural effusion (OR, 1.30; *p* = 0.046) on admission.

Further, the factors that demonstrated a significant negative association with an increase in weakness or lack of energy were modified PiPS-A with predicted prognoses of surviving weeks (OR, 0.72; *p* = 0.014) and days (OR, 0.51; *p* = 0.002) on admission. For worsening drowsiness, only the modified PiPS-A with a predicted prognosis of surviving days (OR, 0.66; *p* = 0.038) on admission was a negatively associated factor. However, we found no significantly associated factors for worsening of pain and sore or dry mouth (Table 3).

**Table 3.** Multivariate Logistic Regression Analysis of Factors Associated With the Worsening of Symptoms During the Period Patients Received Specialist Palliative Care

*Variables*	*Pain*			*Shortness of breath*			*Weakness or lack of energy*			*Sore or dry mouth*			*Drowsiness*		
	*OR*	*95% CI*	****p****	*OR*	*95% CI*	****p****	*OR*	*95% CI*	****p****	*OR*	*95% CI*	****p****	*OR*	*95% CI*	****p****
Unadjusted model															
Palliative care at home	0.79	0.35–1.80	0.541	1.42	1.08–1.88	0.014	1.11	0.73–1.71	0.596	1.00	0.59–1.69	0.986	1.23	0.86–1.78	0.238
Adjusted model															
Palliative care at home	0.75	0.34–1.65	0.436	1.14	0.85–1.52	0.375	0.93	0.63–1.38	0.711	0.90	0.54–1.48	0.641	1.07	0.73–1.58	0.706
Demographic and clinical characteristics at registration															
Age ≥65	0.82	0.49–1.37	0.417	0.87	0.64–1.19	0.374	0.83	0.63–1.10	0.188	0.95	0.72–1.24	0.678	0.96	0.65–1.41	0.824
Male sex	0.93	0.63–1.37	0.696	1.10	0.89–1.35	0.379	0.99	0.79–1.25	0.951	1.14	0.92–1.41	0.226	0.94	0.72–1.23	0.636
Site of primary cancer: Lung	—	—	—	1.48	1.07–2.05	0.020	—	—	—	—	—	—	—	—	—
Site of metastasis: bone	1.02	0.64–1.62	0.924	—	—	—	—	—	—	—	—	—	—	—	—
Site of metastasis: lung	—	—	—	1.41	1.15–1.73	<0.001	—	—	—	—	—	—	—	—	—
Site of metastasis: central nervous system	—	—	—	—	—	—	—	—	—	—	—	—	1.18	0.85–1.65	0.323
Anorexia	—	—	—	—	—	—	0.77	0.52–1.15	0.190	—	—	—	—	—	—
Weight loss in the previous month	—	—	—	—	—	—	0.99	0.77–1.26	0.901	—	—	—	—	—	—
Pleural effusion	—	—	—	1.30	1.01–1.69	0.046	—	—	—	—	—	—	—	—	—
Delirium (DSM-5)	—	—	—	—	—	—	—	—	—	—	—	—	0.90	0.65–1.23	0.484
Modified PiPS-A: months	Ref.	Ref.	Ref.	Ref.	Ref.	Ref.	Ref.	Ref.	Ref.	Ref.	Ref.	Ref.	Ref.	Ref.	Ref.
Modified PiPS-A: weeks	0.83	0.56–1.23	0.329	0.74	0.50–1.09	0.120	0.72	0.56–0.94	0.014	0.82	0.58–1.16	0.243	0.93	0.66–1.32	0.683
Modified PiPS-A: days	0.72	0.43–1.19	0.188	0.47	0.27–0.83	0.012	0.51	0.34–0.77	0.002	0.73	0.45–1.18	0.184	0.66	0.44–0.98	0.038
Palliative Prognostic Index ≥6.5	1.07	0.57–2.00	0.813	0.42	0.31–0.56	<0.001	0.81	0.60–1.11	0.178	0.91	0.69–1.20	0.499	0.90	0.67–1.23	0.512
Age-adjusted Charlson comorbidity index ≥6	1.08	0.54–2.15	0.815	1.08	0.63–1.84	0.778	0.93	0.55–1.57	0.785	0.83	0.46–1.52	0.539	1.11	0.66–1.85	0.692
Opioid dosage (OME ≥60 mg/day)	0.91	0.58–1.43	0.662	1.10	0.73–1.65	0.644	1.05	0.76–1.45	0.774	0.90	0.66–1.24	0.516	0.84	0.58–1.23	0.355
Chemotherapy within a month	—	—	—	—	—	—	0.94	0.72–1.21	0.611	—	—	—	—	—	—
Oxygen therapy use	—	—	—	0.80	0.58–1.10	0.161	—	—	—	0.96	0.77–1.21	0.748	—	—	—
Antipsychotic use	—	—	—	—	—	—	0.85	0.67–1.09	0.194	0.89	0.63–1.24	0.477	0.90	0.66–1.23	0.494
Psychotropic use	—	—	—	0.99	0.77–1.28	0.965	1.04	0.86–1.26	0.711	1.04	0.85–1.29	0.698	1.14	0.92–1.41	0.227
Treatment at one week before death															
Opioid dosage (OME ≥60 mg/day)	0.90	0.61–1.35	0.592	1.03	0.60–1.77	0.916	0.99	0.70–1.38	0.929	1.00	0.68–1.47	0.987	0.94	0.65–1.36	0.745
Parenteral hydration: 0 mL/day	—	—	—	Ref.	Ref.	Ref.	Ref.	Ref.	Ref.	Ref.	Ref.	Ref.	—	—	—
Parenteral hydration: 1–999 mL/day	—	—	—	0.95	0.38–2.37	0.905	0.93	0.68–1.29	0.661	1.01	0.70–1.46	0.961	—	—	—
Parenteral hydration: ≥1000 mL/day	—	—	—	2.59	0.43–15.56	0.262	0.97	0.35–2.66	0.950	0.83	0.34–2.00	0.653	—	—	—

CI, confidence interval; OR, odds ratio.

## Discussion

In this study, the proportion of patients with an increase in pain and drowsiness was significantly higher among the patients treated at home than in those treated in PCUs. However, after adjusting for associated factors by multivariate logistic regression analysis, the prevalence of worsening of these two symptoms no longer showed a significant difference between palliative care at home and PCU groups.

In addition, the prevalence of worsening of shortness of breath, weakness or lack of energy, and sore or dry mouth did not differ significantly between PCUs and palliative care at home. This result was also confirmed by the multivariate logistic regression analysis. The most important finding of this study was that after adjusting for patient backgrounds, such as disease stage, estimated prognosis, symptoms, and treatment, the prevalence of worsening of all five symptoms was not different between patients receiving palliative care at home and in PCUs.

The second important finding was regarding opioid dosage at one week before death. Opioid dosage (OME ≥60 mg/day) was significantly more prevalent in patients admitted to PCUs than in those receiving palliative care at home. Nevertheless, as described earlier, the worsening of all symptoms was not significantly different between PCUs and palliative care at home.

We did not find any studies directly comparing opioid dosage between PCUs and palliative care at home, but several studies compared opioid dosage in different settings. One study revealed that patients admitted to a PCU received higher opioid dosages than those admitted to an oncological ward.^40^ Another study revealed that patients receiving palliative care at home reported having been administered with stable opioid dosages in the last two weeks of life.^41^

These results might indicate that PCUs tend to evaluate symptom intensity more frequently than at home, resulting in the use of higher opioid dosages. These results might also indicate that physicians have different thresholds for intervening in the treatment of patients in PCUs than those at home. Further research, including interview studies of physicians' opioid prescribing behavior, is needed.

### Clinical implications

We believe that the results of this study may cause patients, their families, and health care professionals to reconsider the known barrier of fear of inadequate symptom control when initiating palliative home care.

### Strengths and limitations

This study has several strengths. First, we had a large sample size representative of patients with advanced cancer receiving specialist palliative care. Second, we used multidisciplinary adjustment variables that included patient backgrounds such as disease stage, estimated prognosis, symptoms, and treatments, to overcome the limitations of previous studies.

This study had some limitations. First, we used the PPI dyspnea subscale to assess shortness of breath, but it has not been validated as a symptom rating scale. Given that the IPOS subscale shortness of breath was assessed only on admission, we considered it insufficient when comparing the prevalence of worsening of symptoms. Therefore, in this study, we used both the PPI subscale dyspnea and the IPOS subscale shortness of breath.

Second, we did not investigate all the frequent end-of-life symptoms, such as nausea, vomiting, and constipation. Third, we included clinics that were actively providing palliative care at home, so our study results could not be generalized to all home care services. Fourth, the number of patients excluded because of unknown date of death was higher for those receiving palliative care at home than in PCUs.

Fifth, in determining the place of care, we could not adjust for confounding factors that influence the choice of the place of care and dying; these factors include patient and family preferences and family caregiving capacity. Sixth, sometimes, patients at home were not evaluated on a set date three days before death. Therefore, some of the home assessments were based on physicians' estimates, which may have led to under- or overestimation of symptoms for patients receiving palliative care at home.

Finally, our study was based on the reports of symptoms by physicians. Patient-reported outcomes are the gold standard for assessing symptom prevalence and severity, but data can be difficult to obtain from patients before death due to impaired consciousness and deterioration of their condition. Therefore, to prevent missing data and to ensure that the same method is used on admission and just before death, we decided to adopt the assessment of symptoms by health care professionals.

To overcome these limitations, future research should examine symptom intensity using patient-reported outcome measures and analyze them using confounding factors that influence the choice of place of care and dying (e.g., patient and family preferences and family caregiving capacity).

## Conclusions

After adjusting for patient backgrounds such as disease stage, estimated prognosis, symptoms, and treatments, the prevalence of the worsening of symptoms such as pain, shortness of breath, weakness or lack of energy, sore or dry mouth, and drowsiness was not significantly different between patients with advanced cancer receiving palliative care at home and in PCUs.

## Abbreviations Used

ACCI: age-adjusted Charlson Comorbidity Index
CI: confidence interval
DSM-5: Diagnostic and Statistical Manual of Mental Disorders 5
EASED: East Asian collaborative cross-cultural Study to Elucidate the Dying process
ECOG PS: Eastern Cooperative Oncology Group performance status ()
IPOS: Integrated Palliative Care Outcome Scale
OME: oral morphine equivalent
OR: odds ratio
PCUs: palliative care units
PiPS-A: Prognosis in Palliative Care Study predictor models-A
PPI: Palliative Prognostic Index

## References

[1] Bajwah S, Oluyase AO, Yi D, et al. The effectiveness and cost-effectiveness of hospital-based specialist palliative care for adults with advanced illness and their caregivers. Cochrane Database Syst Rev 2020;9(9):CD012780; doi: 10.1002/14651858.CD012780.PUB2 32996586 PMC8428758

[2] Gomes B, Calanzani N, Curiale V, et al. Effectiveness and cost-effectiveness of home palliative care services for adults with advanced illness and their caregivers. Cochrane Database Syst Rev 2013;2016(6):CD007760; doi: 10.1002/14651858.CD007760.PUB2 PMC4473359 23744578

[3] Kenny P, Street DJ, Hall J, et al. Valuing end-of-life care for older people with advanced cancer: Is dying at home important? Patient 2021;14(6):803–813; doi: 10.1007/S40271-021-00517-Z 33876399

[4] Currow DC, Agar MR, Phillips JL. Role of hospice care at the end of life for people with cancer. J Clin Oncol 2020;38(9):937–943; doi: 10.1200/JCO.18.02235 32023154

[5] Eagar K, Clapham SP, Allingham SF. Palliative care is effective: But hospital symptom outcomes superior. BMJ Support Palliat Care 2020;10(2):186–190; doi: 10.1136/BMJSPCARE-2018-001534 PMC7286033 30171042

[6] Brescia FJ, Adler D, Gray G, et al. Hospitalized advanced cancer patients: A profile. J Pain Symptom Manage 1990;5(4):221–227; doi: 10.1016/0885-3924(90)90015-C 2384701

[7] Curtis EB, Krech R, Walsh TD, et al. Common symptoms in patients with advanced cancer. J Palliat Care 1991;7(2):25–29. 1870042

[8] Grond S, Zech D, Diefenbach C, et al. Prevalence and pattern of symptoms in patients with cancer pain: a prospective evaluation of 1635 cancer patients referred to a pain clinic. J Pain Symptom Manage 1994;9(6):372–382; doi: 10.1016/0885-3924(94)90174-0 7963790

[9] Donnelly S, Walsh D, Walsh D. The symptoms of advanced cancer. Semin Oncol 1995;22(2 Suppl 3):67–72. 7537907

[10] Vainio A, Auvinen A. Prevalence of symptoms among patients with advanced cancer: an international collaborative study. Symptom Prevalence Group. J Pain Symptom Manage 1996;12(1):3–10; doi: 10.1016/0885-3924(96)00042-5 8718910

[11] Miyashita M, Sanjo M, Morita T, et al. Good death in cancer care: A nationwide quantitative study. Ann Oncol 2007;18(6):1090–1097; doi: 10.1093/ANNONC/MDM068 17355953

[12] Gomes B, Calanzani N, Gysels M, et al. Heterogeneity and changes in preferences for dying at home: A systematic review. BMC Palliat Care 2013;12(1):7; doi: 10.1186/1472-684X-12-7 23414145 PMC3623898

[13] Sarmento VP, Gysels M, Higginson IJ, et al. Home palliative care works: but how? A meta-ethnography of the experiences of patients and family caregivers. BMJ Support Palliat Care 2017;7(4):390–403; doi: 10.1136/BMJSPCARE-2016-001141 28232515

[14] Gomes B, Higginson IJ. Factors influencing death at home in terminally ill patients with cancer: Systematic review. BMJ 2006;332(7540):515–518; doi: 10.1136/BMJ.38740.614954.55 16467346 PMC1388126

[15] Gomes B, Calanzani N, Koffman J, et al. Is dying in hospital better than home in incurable cancer and what factors influence this? A population-based study. BMC Med 2015;13(1):235; doi: 10.1186/S12916-015-0466-5 26449231 PMC4599664

[16] Ministry of Health, Labor and Welfare. Survey on Attitudes toward in the End-of-Life Care (In Japanese). n.d. Available from: https://www.mhlw.go.jp/stf/shingi2/0000200742.html [Last accessed: June 8, 2022].

[17] Ministry of Health, Labor and Welfare. Vital Statistics of Japan. The Latest Trends. n.d. Available from: https://www.mhlw.go.jp/english/database/db-hw/vs01.html [Last accessed: June 8, 2022].

[18] The Yuumi Memorial Foundation for Home Health Care. Barriers of Introducing Home Palliative Care in Japan. A Nationwide Survey among Medical Social Workers and Discharge Support Nurses in Government Designated Cancer Hospitals (In Japanese). n.d. Available from: http://118.82.88.171/main/result.php?year=2014&type=1 [Last accessed: June 8, 2022].

[19] Shepperd S, Gonçalves-Bradley DC, Straus SE, et al. Hospital at home: Home-based end-of-life care. Cochrane Database Syst Rev 2021;3(3); doi: 10.1002/14651858.CD009231.PUB3 PMC8092626 33721912

[20] Yamaguchi T, Maeda I, Hatano Y, et al. Communication and behavior of palliative care physicians of patients with cancer near end of life in three East Asian Countries. J Pain Symptom Manage 2021;61(2):315–322.e1; doi: 10.1016/J.JPAINSYMMAN.2020.07.031 32777459

[21] Shinjo T, Shimizu M, Miyake K, et al. Survey of the circumstances of cancer patients treated at home and the presence of doctors and nurses at the time of death. Palliat Care Res 2020;15(4):259–263; doi: 10.2512/jspm.15.259

[22] Sakurai H, Miyashita M, Imai K, et al. Validation of the Integrated Palliative care Outcome Scale (IPOS)—Japanese Version. Jpn J Clin Oncol 2019;49(3):257–262; doi: 10.1093/JJCO/HYY203 30668720

[23] Schildmann EK, Groeneveld EI, Denzel J, et al. Discovering the hidden benefits of cognitive interviewing in two languages: The first phase of a validation study of the Integrated Palliative care Outcome Scale. Palliat Med 2016;30(6):599–610; doi: 10.1177/0269216315608348 26415736 PMC4873725

[24] Hearn J, Higginson IJ. Development and validation of a core outcome measure for palliative care: The palliative care outcome scale. Palliative Care Core Audit Project Advisory Group. Qual Health Care 1999;8(4):219–227; doi: 10.1136/QSHC.8.4.219 10847883 PMC2483665

[25] Sakurai H, Miyashita M, Morita T, et al. Comparison between patient-reported and clinician-reported outcomes: Validation of the Japanese version of the Integrated Palliative care Outcome Scale for staff. Palliat Support Care 2021;19(6):702–708; doi: 10.1017/S1478951521000018 33666153

[26] Morita T, Tsunoda J, Inoue S, et al. The Palliative Prognostic Index: A scoring system for survival prediction of terminally ill cancer patients. Support Care Cancer 1999;7(3):128–133; doi: 10.1007/S005200050242 10335930

[27] Dumitrescu L, van den Heuvel-Olaroiu M, van den Heuvel WJA. Changes in symptoms and pain intensity of cancer patients after enrollment in palliative care at home. J Pain Symptom Manage 2007;34(5):488–496; doi: 10.1016/J.JPAINSYMMAN.2007.05.004 17697762

[28] Lim KH, Nguyen NN, Qian Y, et al. Frequency, outcomes, and associated factors for opioid-induced neurotoxicity in patients with advanced cancer receiving opioids in inpatient palliative care. J Palliat Med 2018;21(12):1698–1704; doi: 10.1089/JPM.2018.0169 30260731 PMC6308282

[29] Mercadante S, Fulfaro F, Casuccio A. The impact of home palliative care on symptoms in advanced cancer patients. Support Care Cancer 2000;8(4):307–310; doi: 10.1007/S005209900110 10923771

[30] Koppie TM, Serio AM, Vickers AJ, et al. Age-adjusted Charlson comorbidity score is associated with treatment decisions and clinical outcomes for patients undergoing radical cystectomy for bladder cancer. Cancer 2008;112(11):2384–2392; doi: 10.1002/CNCR.23462 18404699

[31] Baba M, Maeda I, Morita T, et al. Independent validation of the modified prognosis palliative care study predictor models in three palliative care settings. J Pain Symptom Manage 2015;49(5):853–860; doi: 10.1016/J.JPAINSYMMAN.2014.10.010 25499420

[32] Gwilliam B, Keeley V, Todd C, et al. Development of prognosis in palliative care study (PiPS) predictor models to improve prognostication in advanced cancer: Prospective cohort study. BMJ 2011;343(7821); doi: 10.1136/BMJ.D4920 PMC3162041 21868477

[33] Murtagh FEM, Ramsenthaler C, Firth A, et al. A brief, patient- and proxy-reported outcome measure in advanced illness: Validity, reliability and responsiveness of the Integrated Palliative care Outcome Scale (IPOS). Palliat Med 2019;33(8):1045–1057; doi: 10.1177/0269216319854264 31185804 PMC6691591

[34] Siriwardana AN, Hoffman AT, Brennan FP, et al. Impact of renal supportive care on symptom burden in dialysis patients: A prospective observational cohort study. J Pain Symptom Manage 2020;60(4):725–736; doi: 10.1016/J.JPAINSYMMAN.2020.04.030 32389605

[35] Pedersen AB, Mikkelsen EM, Cronin-Fenton D, et al. Missing data and multiple imputation in clinical epidemiological research. Clin Epidemiol 2017;9:157–166; doi: 10.2147/CLEP.S129785 28352203 PMC5358992

[36] Morita T, Hyodo I, Yoshimi T, et al. Association between hydration volume and symptoms in terminally ill cancer patients with abdominal malignancies. Ann Oncol 2005;16(4):640–647; doi: 10.1093/ANNONC/MDI121 15684225

[37] Wu CC, Hsu TW, Chang CM, et al. Age-adjusted Charlson comorbidity index scores as predictor of survival in colorectal cancer patients who underwent surgical resection and chemoradiation. Medicine 2015;94(2):e431; doi: 10.1097/MD.0000000000000431 25590852 PMC4602551

[38] Tian Y, Jian Z, Xu B, et al. Age-adjusted Charlson comorbidity index score as predictor of survival of patients with digestive system cancer who have undergone surgical resection. Oncotarget 2017;8(45):79453–79461; doi: 10.18632/ONCOTARGET.18401 29108324 PMC5668057

[39] Bruera E, Hui D, Dalal S, et al. Parenteral hydration in patients with advanced cancer: A multicenter, double-blind, placebo-controlled randomized trial. J Clin Oncol 2013;31(1):111–118; doi: 10.1200/JCO.2012.44.6518 23169523 PMC3530688

[40] Mercadante S, Marchetti P, Adile C, et al. Characteristics and care pathways of advanced cancer patients in a palliative-supportive care unit and an oncological ward. Support Care Cancer 2018;26(6):1961–1966; doi: 10.1007/S00520-017-4037-5. 29313129

[41] Mercadante S, Casuccio A, Pumo S, et al. Factors influencing the opioid response in advanced cancer patients with pain followed at home: The effects of age and gender. Support Care Cancer 2000;8(2):123–130; doi: 10.1007/S005200050026 10739359

